# Measuring the Economic Burden of Family Caregiving in a Rapidly Aging East Asian Population

**DOI:** 10.1093/geroni/igaf122.035

**Published:** 2025-12-31

**Authors:** Xingnan Yi, Xuechen Xiong, Vivian Lou, Jianchao Quan

**Affiliations:** The University of Hong Kong, Hong Kong, China (People’s Republic); The University of Hong Kong, Hong Kong, China (People’s Republic); The University of Hong Kong, Hong Kong, China (People’s Republic); The University of Hong Kong, Hong Kong, China (People’s Republic)

## Abstract

Hong Kong’s aging population, with 19.6% age ≥65 in 2021, has increased reliance on unpaid family caregiving to meet long-term care needs. While psychological impacts are well documented, this study quantifies the direct and indirect economic costs of family caregiving. Data were collected from 2,284 adult caregivers age ≥18 who provided at least six hours of care weekly to family members age ≥60 in 2023–2024. Direct costs included caregiving time (valued using the opportunity cost method), out-of-pocket expenses (OOP), and medical costs due to caregiving-related health issues. Indirect costs included income losses from job cessation, reduced work hours, and productivity impairment due to providing caregiving tasks. Lost hours were quantified through self-reported work absences and productivity reductions, with income loss calculated by multiplying total missed hours by wage level using the opportunity cost method. The total annual economic burden of family caregiving in Hong Kong was estimated at USD 6.65 billion, or 2% of GDP. The mean annual monetary value of caregiving time was USD 6,770 per caregiver. OOP expenses, reported by 68% of caregivers, averaged USD 3,867 annually, while medical costs related to caregiving burden, incurred by 11%, averaged USD 2,350. Indirect costs included income losses from job cessation (reported by 44%) at USD 1,077, reduced work hours (56%) at USD 146, and productivity impairment (43%) leading to USD 2,454 in lost income annually per caregiver. These findings highlight the substantial economic value of unpaid caregiving and underscore the urgent need for policy actions to support family caregivers.

